# Sleep-disordered breathing does not impact maternal outcomes in women with hypertensive disorders of pregnancy

**DOI:** 10.1371/journal.pone.0232287

**Published:** 2020-04-27

**Authors:** Danielle L. Wilson, Mark E. Howard, Alison M. Fung, Fergal J. O’Donoghue, Maree Barnes, Martha Lappas, Susan P. Walker

**Affiliations:** 1 Institute for Breathing and Sleep, Austin Health, Heidelberg, Victoria, Australia; 2 Mercy Perinatal, Mercy Hospital for Women, Heidelberg, Victoria, Australia; 3 Department of Medicine, University of Melbourne, Parkville, Victoria, Australia; 4 Department of Obstetrics and Gynaecology, University of Melbourne, Parkville, Victoria, Australia; University of Mississippi Medical Center, UNITED STATES

## Abstract

**Objective:**

Sleep-disordered breathing (SDB) is characterised by intermittent hypoxemia, sympathetic activation and widespread endothelial dysfunction, sharing pathophysiologic features with the hypertensive disorders of pregnancy. We sought to determine whether coexisting SDB would adversely impact the outcomes of women with gestational hypertension (GH) and preeclampsia (PE), and healthy matched controls.

**Study design:**

Women diagnosed with GH or PE along with BMI- and gestation-matched normotensive controls underwent polysomnography in late pregnancy to establish the presence or absence of SDB (RDI ≥ 5). Clinical outcomes of hypertensive disease severity were compared between groups, and venous blood samples were taken in the third trimester and at delivery to examine for any impact of SDB on the anti-angiogenic markers of PE.

**Results:**

Data was available for 17 women with PE, 24 women with GH and 44 controls. SDB was diagnosed in 41% of the PE group, 63% of the GH group and 39% of the control group. Women with PE and co-existing SDB did not have worse outcomes in terms of gestation at diagnosis of PE (SDB = 29.1 (25.9, 32.1) weeks vs. no SDB = 32.0 (29.0, 33.9), p = n.s.) and days between diagnosis of PE and delivery (SDB = 20.0 (4.0, 35.0) days vs. no SDB = 10.5 (9.0, 14.0), p = n.s.). There were also no differences in severity of hypertension, antihypertensive treatment and biochemical, haematological and anti-angiogenic markers of PE between SDB and no SDB groups. Similar results were observed among women with GH. Healthy control women with SDB were no more likely to develop a hypertensive disorder of pregnancy in the later stages of pregnancy (SDB = 5.9% vs. no SDB = 7.4%, p = n.s.). Increasing the threshold for diagnosis of SDB to RDI ≥ 15 did not unmask a worse prognosis.

**Conclusion:**

The presence of SDB during pregnancy did not worsen the disease course of GH or PE, and was not associated with high blood pressure or anti-angiogenic markers of hypertensive disease amongst healthy pregnant women. Given the numerous reports of the relationship between SDB and diagnosis of hypertensive disorders of pregnancy, it appears more work is required to distinguish causal, versus confounding, pathways.

## Introduction

Preeclampsia (PE) is a serious multi-system disorder that represents a significant threat to the life of the baby and the mother. [1] Limited treatment options have driven a search for potentially modifiable contributors to disease progression. Gestational hypertension (GH) is the main component of PE and up to 1 in 4 women with GH go on to develop PE. In recent years, sleep-disordered breathing (SDB) has been shown to be more common in women with hypertensive disorders of pregnancy (HDP) [2] but only a few experimental studies have attempted to identify the pathophysiology underpinning this relationship. [3,4]

SDB includes a spectrum of sleep-related breathing disorders ranging from snoring to obstructive sleep apnoea (OSA) that are characterised by increased upper airway resistance or obstruction, cessation of airflow and consequent falls in blood oxygen saturation. Outside of pregnancy, SDB confers a 3-fold increase in risk of hypertension independent of other risk factors, [5,6] likely due to the pathophysiological sequelae of sympathetic activation, widespread inflammation and endothelial dysfunction. [7]

Reports regarding the increased frequency of SDB among women with HDP have variably accounted for obesity. [8,9] Given that obesity is a well-recognised risk factor for both hypertension and SDB, our recent work highlighted the importance of adjusting for BMI. [10] What remains unknown however, is how the relationship between SDB and hypertension in pregnancy manifests, and whether there is an independent effect of co-existing SDB on pregnancy outcomes among women with GH or PE. If so, screening and treatment of SDB might have potential as an intervention to prevent or attenuate the severity of HDP, and thus improve perinatal outcomes.

Among pregnant women with SDB, increased sympathetic activity in response to hypoxaemia and hypercapnia may increase peripheral vascular tone, leading to elevations in systemic arterial BP. [11–13] SDB also generates inflammation, oxidative stress, and the release of other factors that could contribute to placental dysfunction. Subsequently, the release of anti-angiogenic proteins from the pathological placenta generate widespread endothelial dysfunction, characteristic of PE. [14] The recurrent episodic hypoxia and reoxygenation of SDB may further worsen placental ischaemia, impacting on the release of these proteins and subsequent damage to the maternal endothelium. It is therefore plausible that the effects of SDB may amplify the negative consequences of both GH and PE through similar pathological pathways. As SDB is treatable, this would provide a new therapeutic avenue for HDP.

The aims of this study were two-fold. Firstly, we hypothesised that co-existing SDB would negatively impact the disease course of women with GH and PE, as evidenced by earlier diagnosis of GH or PE and earlier delivery, poorer BP control, increased anti-hypertensive requirements and worsening biochemical and angiogenic markers of disease. Secondly, we aimed to see whether the presence of SDB predisposes healthy women to the development of HDP in later pregnancy.

## Method

### Study participants

This study was approved by the Human Research Ethics Committees at Austin Health (H2012/04469), Mercy Hospital for Women (R12/02) and University of Melbourne in Melbourne, Victoria, Australia. All subjects gave written informed consent to participate in the study. The data presented here is a secondary analysis of Wilson et al. [10] which was a matched case-control study comparing the prevalence of SDB in women diagnosed with PE and GH to women with normotensive pregnancies.

As per our initial study, [10] women diagnosed with GH or PE between 26 and 37 weeks gestation were considered as cases. Control participants were normotensive women with an uncomplicated pregnancy and were one-to-one matched by BMI to each of the hypertensive cases (within ±4kg/m^2^, measured at the first antenatal appointment). Cases were recruited from the Pregnancy Day Assessment Centre or as inpatients, whereas controls were recruited from the antenatal outpatients clinic. Exclusion criteria for cases and controls included <18 years of age, multiple gestation, fetal abnormality or any non-HDP maternal/fetal condition likely to mandate early or imminent delivery, and previous diagnosis of a sleep disorder.

Hypertension in pregnancy, and the diagnosis of GH and PE were defined as per the most recent International Society for the Study of Hypertension in Pregnancy (ISSHP) statement. [15] Women with chronic hypertension who developed superimposed PE were also eligible to participate as a PE case. All cases of GH or PE were reviewed by a senior obstetrician blinded to SDB status to confirm the diagnosis.

### Procedure

To establish the presence or absence of SDB, participants with HDP underwent full overnight polysomnography (PSG, ‘sleep study’) at their earliest convenience. Control women underwent PSG within ±4 weeks of gestational age to their matched hypertensive case participant. To enhance recruitment, participants were given the choice of attended overnight PSG conducted in the Austin Health sleep laboratory (Compumedics E series—Abbotsford, Victoria, Australia), or unattended PSG in the participant’s home (Somté portable sleep monitoring device—Compumedics). Inpatients were studied using the portable device. PSG recordings were analysed using the American Academy of Sleep Medicine (AASM) criteria, [16] by an experienced sleep technologist who was blinded to all participant details. The respiratory disturbance index (RDI) was calculated as the number of apneas and/or hypopneas and/or RERAs per hour of sleep. SDB was defined as an RDI of ≥5 events per hour, with secondary analyses performed with SDB defined as RDI ≥15. SDB severity is classified as mild (RDI 5–14.9/hr), moderate (RDI 15–29.9/hr) and severe (RDI ≥30) in sleep literature. [17] The oxygen desaturation index (ODI ≥3%) was defined as the number of arterial oxygen desaturations of ≥3% from baseline, per hour of sleep. [16]

Within ± three weeks of the PSG a venous blood sample was taken from each participant, and another was taken when admitted for delivery. Each sample was assayed for endothelin-1 (ET-1), soluble fms-like tyrosine kinase-1 (sFlt-1), soluble endoglin (sEng) and placental growth factor (PlGF) levels in maternal plasma, and were measured using Quantikine ELISA kits (R&D Systems, Minneapolis, USA). Intra- and interassay coefficients of variation were less than 10% and quality control values were within the specified ranges.

Baseline demographic information as well as relevant comorbidities such as diagnosis of gestational diabetes mellitus (GDM) [18] were recorded. After delivery, medical records were reviewed for the relevant outcome data, including gestation at diagnosis of GH or PE, days between diagnosis and delivery of the fetus, and use of anti-hypertensive medication. Hypertension was considered to be severe if BP was >160mmHg systolic and/or >110mmHg diastolic on more than one occasion, and PE was considered as early-onset if diagnosed prior to 34 weeks gestation. [19] BP recorded at each antenatal appointment was also collected. Participants with suspected or confirmed hypertension in pregnancy were routinely sent for blood sampling to measure biochemical indicators of PE severity.

### Statistical analysis

GH and PE have different pathologic, pathogenetic and hemodynamic characteristics, [20–23] thus SDB may affect the course of GH and PE differently. For these reasons, the effect that SDB has on the clinical outcomes of control women, and those with GH and PE was considered separately (apart from analysis of maternal blood markers due to missing data).

All statistical analyses were performed with SPSS 21.0 (SPSS Inc., Chicago, Illinois). For consistency, values are given in median and interquartile range (*Mdn (IQR)*) due to the small sample size in the PE group, and as some variables were non-normally distributed. A two-sided p value of less than .05 was considered to indicate statistical significance. SDB was defined as an RDI of ≥5 events per hour, with supplementary analyses performed with SDB defined as RDI ≥15 to investigate the impact of more severe SDB on HDP.

Comparisons between the SDB and No SDB groups were done using Fisher’s exact test of independence for categorical variables and Mann-Whitney U tests for continuous variables. A sequential Cox regression survival analysis was performed to test the hypothesis that women with SDB will have a shorter gestation between diagnosis of a hypertensive disorder and delivery, than those without SDB, after adjusting for the effects of covariates with a p value of less than 0.10 on the log rank test.

Mixed modelling was conducted to investigate change in systolic and diastolic BP across pregnancy for those with and without SDB, taking into account the risk factors and treatment for hypertension in pregnancy. BP measurements were noted from each antenatal appointment that was closest to specific gestations (as shown in Results) for each group. Analysis was capped at the gestation after which more than half of the participants in that group had delivered. Longitudinal BP data was not available for five control participants and one PE participant. Forward stepwise selection was used to construct a model using SDB and gestation as fixed factors and anti-hypertensive use at each gestation as a covariate. Other covariates tested in the model included BMI at first antenatal appointment, gestational weight gain, GDM, age, and parity; the explanatory variable with the smallest p value (of less than 0.20) was added at each step.

Maternal blood samples taken at the time of the sleep study were referred to as the ‘third trimester’ sample and the second sample taken at delivery referred to as the ‘delivery’ sample. As the maternal blood samples were taken at different gestations for each participant, a rate of change per week from the third trimester sample to the delivery sample was calculated.

## Results

### Demographics

A total of 87 pregnant women participated, with one sleep study failure in the control group and one control participant excluded due to change in care provider. As per Table 1, 41% of the PE group, 63% of the GH group and 39% of the control group had SDB (p = .15). Within each group, there were no differences in age, parity, GDM, BMI, gestational weight gain and gestation at sleep study for the SDB versus No SDB groups.

**Table 1:** Demographics for preeclampsia and gestational hypertension cases and normotensive control groups stratified by SDB status.

	PE (n = 17)			GH (n = 24)			Controls (n = 44)		
	SDB (n = 7)	No SDB (n = 10)	p	SDB (n = 15)	No SDB (n = 9)	p	SDB (n = 17)	No SDB (n = 27)	P
Age (years)	31.0 (30.0, 34.0)	33.5 (26.0, 35.5)	.38	36.0 (29.0, 38.0)	33.0 (29.0, 34.0)	.28	33.0 (31.5, 36.0)	33.0 (29.0, 38.0)	.95
Nulliparous	6 (85.7%)	7 (70.0%)	.60	8 (53.3%)	5 (55.6%)	1.0	10 (58.8%)	13 (48.1%)	.55
GDM	3 (41.2%)	3 (30.0%)	.64	2 (13.3%)	0 (0.0%)	.51	4 (23.5%)	4 (14.8%)	.69
BMI first appt*	32.9 (26.5, 40.3)	27.0 (25.4, 29.4)	.24	33.9 (30.5, 36.3)	31.0 (28.1, 33.0)	.16	35.1 (27.4, 38.5)	29.5 (27.7, 36.1)	.47
Gestational weight gain (kg)*	11.0 (5.3, 14.0)	7.5 (3.0, 10.4)	.49	11.0 (9.0, 16.5)	12.0 (5.0, 12.8)	.49	10.0 (6.0, 12.6)	9.4 (5.5, 12.4)	.72
BMI PSG	38.3 (29.4, 46.3)	31.0 (30.1, 31.8)	.31	38.0 (36.4, 40.2)	35.3 (32.3, 38.4)	.11	38.2 (31.6, 42.0)	35.6 (31.2, 37.5)	.28
Gestation PSG (weeks)	29.6 (27.6, 34.4)	32.4 (29.1, 34.0)	.41	35.9 (34.4, 36.7)	35.9 (32.6, 37.6)	.74	33.3 (32.0, 34.9)	34.0 (30.7, 34.6)	.98
RDI/hr	16.8 (12.3, 108.6)	1.9 (1.5, 3.0)	< .001	12.5 (7.2, 26.1)	3.0 (1.1, 3.2)	< .001	8.8 (7.2, 27.3)	2.9 (1.5, 4.0)	< .001
ODI ≥ 3% overall	6.0 (0.4, 109.7)	0.7 (0.2, 1.4)	.055	5.9 (3.2, 35.9)	1.3 (0.0, 2.9)	.002	5.4 (0.7, 27.1)	1.0 (0.2, 2.4)	.007

Values given as Mdn (IQR) or n (%). PE = preeclampsia, GH = gestational hypertension, SDB = sleep-disordered breathing, GDM = gestational diabetes mellitus, BMI = body mass index, kg/m^2^, PSG = polysomnography, RDI = respiratory disturbance index, ODI = oxygen desaturation index.

*The BMI value measured at the first antenatal appointment was taken at a mean of 15.1 ± 2.6 weeks gestation, with gestational weight gain taken from booking until PSG gestation.

Participants had a preference for sleep-monitoring in their homes, with 57% of the controls and 85% of the women with a hypertensive disorder utilising this option. However, there was no difference in the RDI based on whether the sleep study was performed in-laboratory or at home (4.6 [3.7, 12.0] vs. 4.3 [2.0, 10.1], p = .25, respectively), After undergoing PSG, three participants (1 PE, 2 controls) were diagnosed with severe SDB and commenced treatment (Continuous Positive Airway Pressure–CPAP). The PE participant was thus removed from the analysis regarding time from diagnosis of PE to delivery. All three participants were included in the longitudinal analysis of BP however the BP values after commencing CPAP were censored, and they were excluded from the maternal blood marker analysis.

### Sleep-disordered breathing and markers of HDP disease severity

#### Preeclampsia

**Fig 1:**
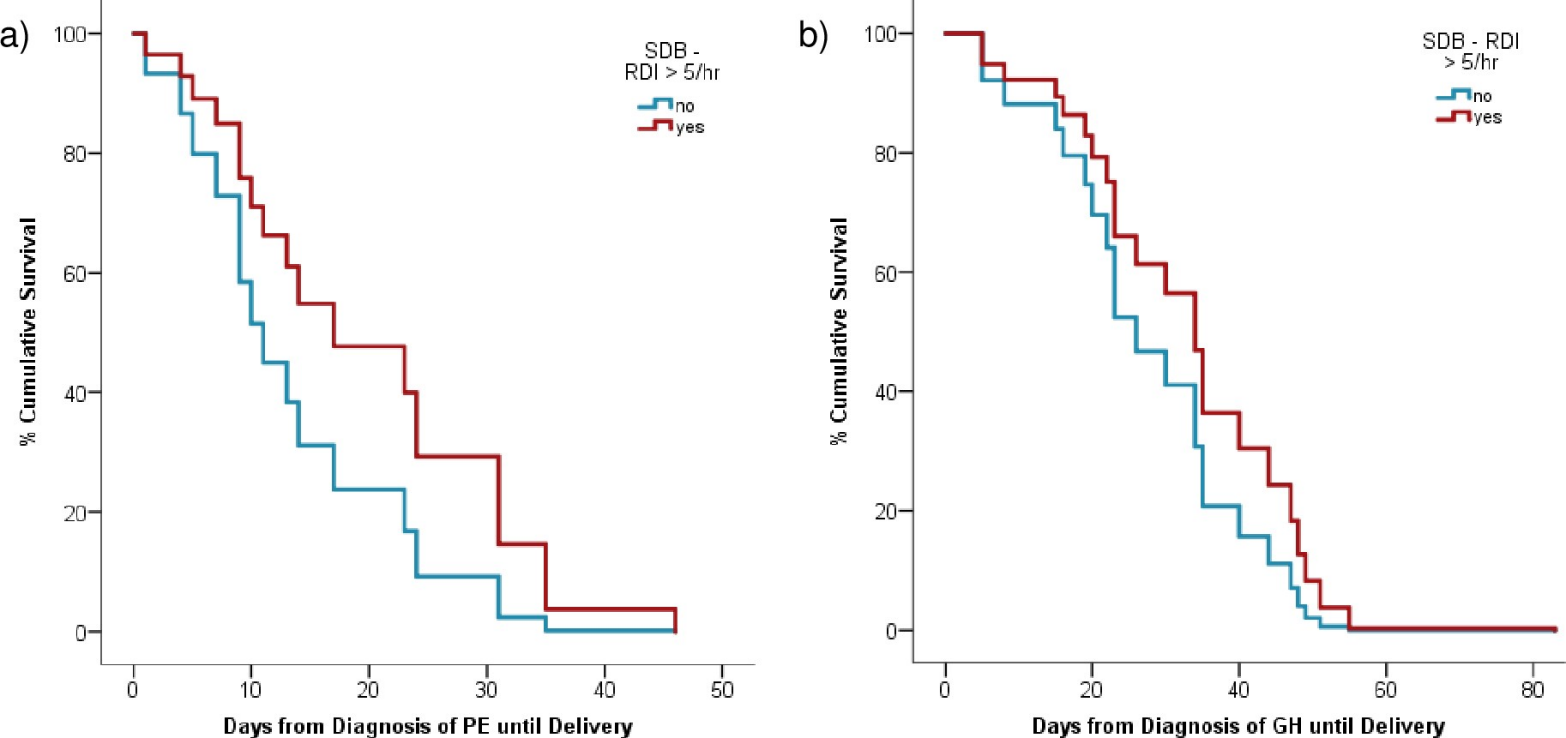
a) Survival curve for the number of days from diagnosis of preeclampsia (PE) until delivery, with the covariate of fetal growth restriction (FGR), for the SDB (n = 6) and No SDB (n = 10) groups. SDB = sleep-disordered breathing, RDI = respiratory disturbance index. b) Survival curve for the number of days between diagnosis of gestational hypertension (GH) and delivery with gestation of diagnosis of GH as a covariate, for the SDB (n = 15) and No SDB (n = 9) groups. SDB = sleep-disordered breathing, RDI = respiratory disturbance index.

As shown in Table 2, there were no differences in the average gestation at diagnosis of PE or at delivery for those with and without SDB. Given this, we used another index of disease severity: the number of days between diagnosis of PE and delivery. The survival curve (Fig 1A) demonstrates that the SDB group had 20.0 (4.0, 35.0) days from diagnosis to delivery compared to 10.5 (9.0, 14.0) days for the No SDB group (p = .51). After adjusting for potential covariates that might impact on preterm birth, particularly coexisting fetal growth restriction (FGR), SDB still had no statistically significant effect on the number of days between PE diagnosis and delivery, X^***2***^ (1) = 1.17, OR 0.52, 95% CI [0.16, 1.71], p = .28, Fig 1A.

**Table 2:** Indices of severity of hypertensive disease for each group stratified by SDB status.

	PE (n = 17)			GH (n = 24)			Controls (n = 44)		
	SDB (n = 7)	No SDB (n = 10)	p	SDB (n = 15)	No SDB (n = 9)	P	SDB (n = 17)	No SDB (n = 27)	p
Gest Diag (weeks)	29.1 (25.9, 32.1)	32.0 (29.0, 33.9)	.28	33.6 (32.1, 34.3)	34.0 (30.7, 35.6)	.93	-	-	
Gest Delivery (weeks)*	31.6 (27.9, 37.1)	33.9 (29.7, 36.4)	.74	37.9 (37.0, 38.9)	38.1 (36.9, 38.6)	.79	39.1 (38.4, 40.3)	39.4 (38.4, 40.7)	.82
Early Onset (<34 w)	6 (85.7%)	8 (80.0%)	1.0	-	-		-	-	
FGR with diag	4 (57.1%)	6 (60%)	1.0	-	-		-	-	
Severe HTN^*#*^	4 (57.1%)	8 (80.0%)	.59	4 (26.7%)	4 (44.4%)	.41	0 (0%)	0 (0%)	-
% Antihypertensive	5 (71.4%)	9 (90.0%)	.54	12 (80.0%)	8 (88.9%)	1.0	0 (0%)	1 (3.7%)	-
>1 Antihypertensive	2 (28.6%)	8 (80.0%)	.06	2 (13.3%)	1 (11.1%)	1.0	-	-	
Gest Antihypert	27.9 (24.7, 32.1)	32.3 (27.3, 34.0)	.29	35.4 (32.8, 37.1)	33.1 (29.9, 36.6)	.28	-	-	
Developed PE	-	-		5 (33.3%)	3 (33.3%)	1.0	0 (0%)	0 (0%)	-
Developed GH	-	-		-	-		1 (5.9%)	2 (7.4%)	.85
*Biochemical and Haematological Markers*									
Peak Pr:Cr Ratio	.10 (.05, .31)	.15 (.05, .38)	.81	.03 (.02, .08)	.02 (.02, .03)	.12	-	-	
Peak ALT	36.0 (15.0, 63.0)	23.0 (13.0, 29.8)	.36	24.0 (15.0, 32.0)	20.0 (16.0, 42.5)	.91	-	-	
Peak Urate	0.46 (0.28, 0.54)	0.49 (0.35, 0.55)	.70	0.34 (0.31, 0.38)	0.33 (0.32, 0.35)	.93	-	-	
Peak Creatinine	65.0 (59.0, 89.0)	68.0 (63.0, 81.0)	.85	57.0 (49.0, 63.0)	64.0 (63.0, 66.0)	.14	-	-	
Nadir Platelets	187.0 (171.0, 241.0)	191.0 (142.0, 216.0)	.70	192.0 (174.0, 258.0)	170.0 (154.0, 218.0)	.26	-	-	

Values given as Mdn (IQR) or n (%). SBD = sleep-disordered breathing, RDI = respiratory disturbance index, PE = preeclampsia, GH = gestational hypertension, gest = gestation, diag = diagnosis, HTN = hypertension, Antihypert = antihypertensive, Pr:Cr = protein:creatinine, ALT = alanine transaminase.

*Three CPAP users removed from analysis for this outcome.

^*#*^ defined as systolic BP ≥ 160mmHg and/or diastolic BP ≥ 110mmHg

As shown in Table 2, SDB did not adversely impact on indices of disease severity among women with PE, including hypertensive disease severity, development of early onset PE (<34 weeks), whether co-existing FGR was present, if hypertension was classified as severe by the end of pregnancy, nor the need for antihypertensive treatment. Multiple anti-hypertensives tended to be administered more often in the No SDB compared to the SDB group. Regarding the peak biochemical and haematological markers of disease severity, there were also no differences observed between those who had SDB and those who did not (Table 2).

**Fig 2:**
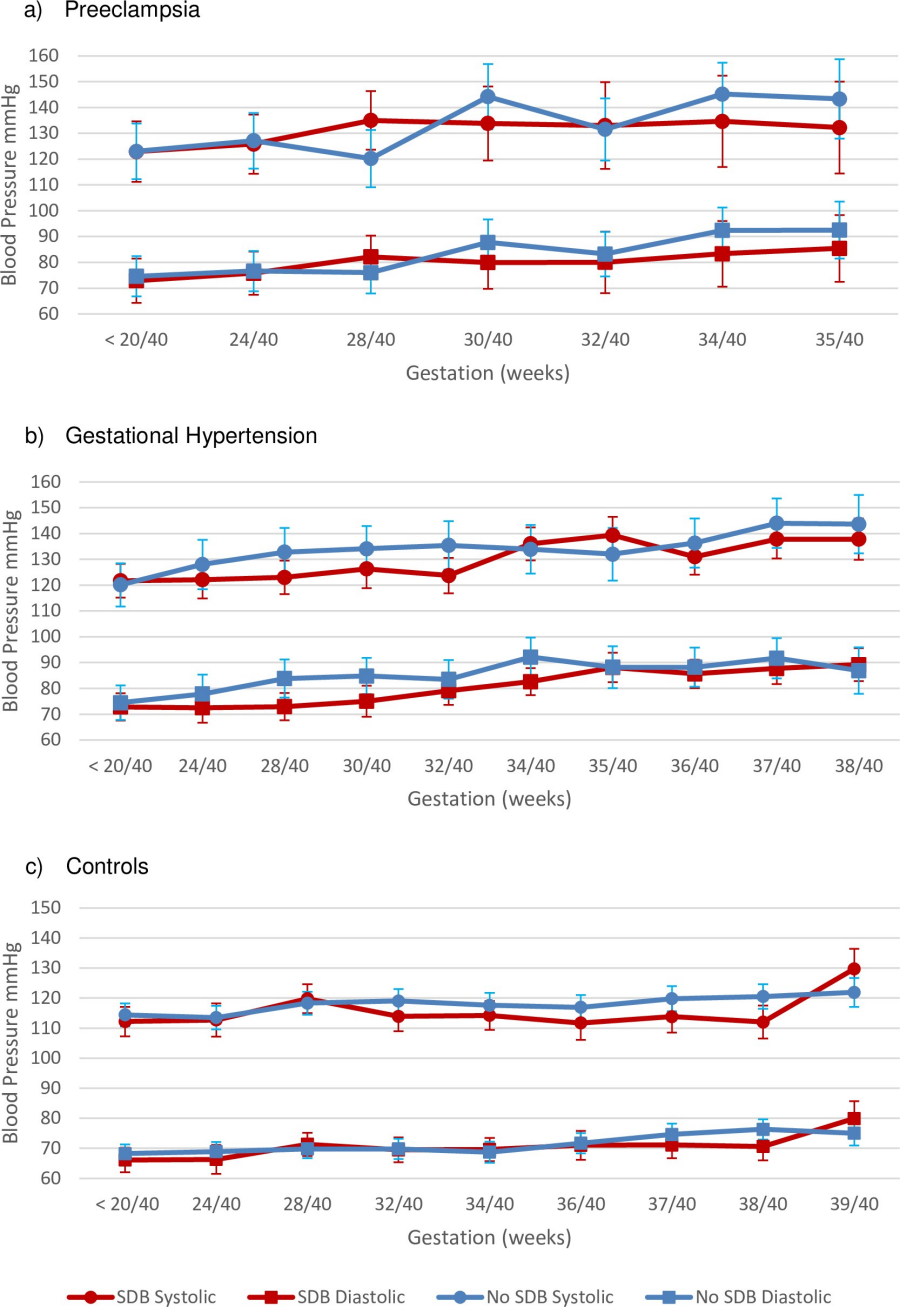
Mean with 95% confidence intervals for systolic and diastolic blood pressure across pregnancy. SDB = sleep-disordered breathing. a) SDB (n = 7) and No SDB (n = 9) within the preeclampsia group. Values based on parameter estimates for the autoregressive mixed model with covariate of antihypertensive use. b) SDB (n = 15) and No SDB (n = 9) within the gestational hypertension (GH) group. Values based on parameter estimates for the mixed model with covariates of antihypertensive use and gestational weight gain for systolic BP, and antihypertensive use, parity and BMI for diastolic BP. c) SDB (n = 15) and No SDB (n = 24) within the control group. Values based on parameter estimates for the mixed model with covariate of BMI for systolic BP, and BMI and age for diastolic BP.

Mixed modelling was conducted to investigate change in BP across pregnancy for those with and without SDB, taking into account risk factors and treatment for hypertension. Within the PE group (Fig 2A), systolic BP rose by 14.8mmHg (95% CI [0.5, 30.0]) on average across pregnancy (p = .11) and diastolic BP rose by 15.2mmHg (95% CI [4.2, 26.2], p = .10). There was no effect of SDB on systolic or diastolic BP (p = .63 and p = .40 respectively) and no interaction between SDB and gestation (systolic p = .15, diastolic p = .42). Anti-hypertensive use was a significant factor; when medication was administered systolic BP was estimated as 12.9mmHg (95% CI [2.72, 23.0] higher than when not required (p = .01).

#### Gestational hypertension

There was no difference in the average gestation at diagnosis of GH or at delivery for those with and without SDB (Table 2). Fig 1B shows the survival curve for the number of days between diagnosis of GH and delivery, showing that on average the SDB group had 30.0 (15.0, 48.0) days from diagnosis to delivery compared to 34.0 (20.0, 44.0) days for the No SDB group (p = .98). After adjusting for gestational age at GH diagnosis, SDB still had no statistically significant effect on the number of days between GH diagnosis and delivery, X^***2***^ (1) = 0.93, OR 0.64, 95% CI [0.26, 1.58], p = .34.

As seen in Table 2, SDB had no effect on the proportion of GH participants who went on to develop PE following their sleep study. Other measures of hypertension were not impacted by SDB (severity of hypertension, use and gestational age at commencement of antihypertensive medication). Furthermore, there were no differences in the biochemical and haematological markers of hypertensive disease severity for the GH group, between those who had SDB and those who did not.

Within the mixed model for longitudinal blood pressure for the GH group (Fig 2B), both systolic and diastolic BP increased significantly across pregnancy (increase of 19.4mmHg, 95% CI [10.3, 28.5], p < .001 and 14.4mmHg, 95% CI [7.1, 21.7], p < .001 respectively). Average systolic BP was significantly lower in the SDB group compared to the no SDB group (129.9mmHg, 95% CI [127.4, 132.3] vs 134.1mmHg, 95% CI [130.8, 137.4], p = .047), as was diastolic BP (80.5 mmHg, 95% CI [78.2, 82.9] vs 85.1mmHg, 95% CI [81.9, 88.4], p = .03). BP was not differentially affected across gestation between the SDB groups (interaction for systolic BP p = .49, diastolic BP p = .56). Parity was also a significant predictor in the model–nulliparous women had an estimated increase of 4.6mmHg (95% CI [0.8, 8.4]) in diastolic BP compared to multiparous women (p = .02).

#### Normotensive controls

Of the 44 women who were normotensive at the time of the sleep study, only three were later diagnosed with mild GH and none developed PE (Table 2). There was no difference in gestational age at delivery between the SDB and no SDB group. Only one participant (in the No SDB group) used antihypertensive medication, administered during labour.

Within the control group, both systolic and diastolic BP increased significantly across pregnancy (increase of 12.5mmHg, 95% CI [7.4, 17.7], p < .001 and increase of 10.3mmHg (95% CI [6.0, 14.6], p < .001 respectively; Fig 2C). There was no effect of SDB on systolic (p = .15) or diastolic BP overall (p = .44). BMI at the first antenatal visit was a significant covariate, with an average increase in systolic BP of 0.42mmHg (95% CI [0.18, 0.66], p = .001) and diastolic BP of 0.49mmHg (95% CI [0.29, 0.68], p < .001) per unit of BMI.

### Anti-angiogenic markers of hypertensive disease

As shown in Table 3, the HDP and control cohorts showed no difference in ET-1, sEng, sFlt-1, or PlGF between the SDB and No SDB group in the third trimester or at delivery. Within the HDP group, there was also no effect of SDB on change per week of gestation for each of these markers. Although there was a slight trend for an increase in sFlt-1 and sEng per week among those with no SDB in the control group, this was not statistically significant (p = .052). The ratio of sFlt-1 to PlGF, which is a marker of imbalance between angiogenic and anti-angiogenic factors and predictive of PE, [24] was not different across SDB groups. The gestational age at which the third trimester sample was taken and the number of days between the third trimester and delivery samples did not differ based on SDB status.

**Table 3:** Third trimester, delivery and change per week for anti-angiogenic markers of hypertensive disease in pregnancy.

	HDP (n = 23)*			Controls (n = 24)		
	SDB (n = 13)	No SDB (n = 10)	p	SDB (n = 8)	No SDB (n = 16)	p
Gestation T3 sample (weeks + days)	35+1 (34+1, 36+6)	36+1 (35+0, 37+4)	.19	32+6 (32+0, 34+3)	34+1 (33+0, 34+6)	.26
Days between samples	13.0 (8.0, 25.0)	8.0 (5.0, 15.0)	.13	41.5 (25.8, 47.8)	37.0 (29.5, 53.3)	.98
*Third Trimester*						
ET-1 (pg/ml)	1.4 (0.9, 1.8)	1.6 (1.3, 2.1)	.28	1.9 (1.4, 3.2)	1.9 (1.1, 2.5)	.45
sEng (ng/ml)	17.9 (6.5, 30.1)	20.4 (13.0, 37.2)	.65	5.6 (2.6, 17.3)	7.1 (3.9, 8.5)	.83
sFlt-1 (pg/ml)	6157.0 (3213.5, 8652.2)	4957.4 (2604.4, 10081.5)	.93	769.4 (487.8, 3623.3)	1050.4 (523.8, 2110.0)	.79
PlGF (pg/ml)	85.2 (59.6, 153.5)	86.9 (47.1, 109.3)	.56	201.9 (82.5, 409.0)	168.6 (125.9, 228.9)	.98
sFlt-1/PlGF	86.1 (29.0, 117.6)	82.6 (29.1, 183.1)	.80	5.1 (1.6, 32.1)	5.2 (2.8, 13.4)	.81
*Delivery*						
ET-1 (pg/ml)	1.6 (1.0, 2.2)	1.9 (1.4, 2.4)	.61	2.8 (1.9, 3.3)	2.2 (1.3, 2.9)	.14
sEng (ng/ml)	15.5 (7.8, 35.9)	23.6 (12.5, 37.5)	.56	6.0 (2.9, 22.1)	10.7 (7.8, 21.2)	.21
sFlt-1 (pg/ml)	7653.9 (4972.4, 9414.6)	5942.6 (2816.5, 11380.3)	.65	2350.0 (1129.1, 5107.1)	3676.0 (2692.4, 5197.5)	.35
PlGF (pg/ml)	81.8 (45.9, 108.0)	77.4 (47.1, 94.0)	.65	144.8 (59.2, 223.5)	89.0 (51.4, 123.0)	.32
sFlt-1/PlGF	103.1 (47.7, 169.2)	87.7 (38.8, 375.3)	.76	27.7 (6.3, 87.5)	45.5 (27.5, 68.3)	.52
*Change per week*						
ET-1 (pg/ml)	0.11 (-0.15, 0.31)	0.05 (-0.30, 0.61)	.78	0.09 (0.02, 0.21)	0.05 (-0.04, 0.14)	.29
sEng (ng/ml)	0.6 (-0.8, 4.2)	-0.3 (-1.7, 2.9)	.74	0.2 (-0.2, 1.0)	0.8 (0.5, 1.9)	.052
sFlt-1 (pg/ml)	553.3 (96.8, 1228.8)	872.6 (-379.1, 1252.9)	.83	204.6 (151.1, 369.2)	459.6 (313.0, 750.8)	.052
PlGF (pg/ml)	-7.1 (-22.7, 1.9)	-4.5 (-12.3, 0.9)	.65	-10.3 (-29.3, -3.4)	-17.9 (-35.9, -8.1)	.26
sFlt-1/PlGF	7.1 (0.5, 24.7)	10.3 (2.6, 41.4)	.62	3.0 (0.8, 9.9)	7.8 (2.9, 11.6)	.30

Values given as Mdn (IQR). HDP = hypertensive disorders of pregnancy, SDB = sleep-disordered breathing, T3 = third trimester, ET-1 = endothelin-1, sEng = soluble endoglin, sFlt-1 = soluble fms-like tyrosine kinase-1, PlGF = placental growth factor.

Three participants on CPAP excluded.

*One PE participant was excluded from analysis due to extreme outlying values (z scores > 5.3).

### Severity of SDB and hypertensive disorders of pregnancy

All the above findings were unchanged even when examined by more severe SDB type (diagnostic threshold raised to an RDI ≥ 15; see S1 and S2 Tables), apart from a significantly later gestation at delivery amongst those with moderate-to-severe SDB in the PE group. Nevertheless, it should be noted that the numbers in each group with at least moderate SDB were very small (4 for PE, 5 for GH, 6 for controls).

## Discussion

This study focused on the impact of objectively measured SDB on numerous indices of hypertensive disease during pregnancy. This is in contrast to previous studies, which have largely described only significant associations between these disease states. Given the commonalities in pathophysiology, we wished to explore whether coexisting SDB confers a worse prognosis in HDP, potentially due to a ‘double hit’ of sympathetic activation, widespread inflammation and endothelial dysfunction. We gathered multiple, clinically relevant indices of disease severity, and carefully adjusted for the impact of confounding variables. Firstly, we found that pregnant women diagnosed with a hypertensive disease and coexisting SDB did not have a worse prognosis, in terms of gestation at diagnosis, diagnosis to delivery interval, severity of hypertension or biochemical, haematological or circulating biomarker measures of disease severity. Secondly, we showed in a sample of normotensive healthy pregnant women that the presence of SDB did not worsen blood pressure or anti-angiogenic markers of hypertensive disease later in pregnancy.

### Preeclampsia development and the potential impact of sleep-disordered breathing

There is substantial cross over between the pathophysiological mechanisms associated with SDB and the pathogenesis of PE. The development of PE is hypothesized to be in two distinct phases, both of which could hypothetically be augmented by SDB. The first stage is reduced placental perfusion caused by errors in vascular remodeling. Failure of cytotrophoblasts to fully invade and switch to adhesion molecules can be reproduced in vitro under hypoxic conditions. [25] At this early stage of pregnancy, it could be hypothesised that women with pre-existing SDB experiencing nocturnal hypoxia could be on an early pathway to developing PE.

During the second phase of PE development, the under-perfused placenta releases anti-angiogenic factors, inflammatory cytokines and markers of oxidative stress into the circulation, triggering maternal endothelial dysfunction [26,27] which leads to hypertension, [26,28] renal insufficiency and proteinuria, liver dysfunction and cerebral edema. [14] Similarly, SDB may propagate vascular endothelial dysfunction through a number of pathways such as hypoxemia, reactive oxygen species production and sympathetic activation. [29] Impaired endothelial function in patients with SDB has been assessed in a number of ways, including as a muted cerebrovascular blood flow response to hypoxia, [30] and as an increased level of circulating apoptotic endothelial cells as a direct marker of endothelial damage. [31] At the second stage of PE development, this pathological sequela of SDB could heighten the maternal endothelial damage, increasing susceptibility of the vasculature to the effects of circulating anti-angiogenic factors, [32] thus worsening tissue damage. Compounding the damage to the already ischemic placenta, intermittent hypoxia from SDB could further induce the release of anti-angiogenic factors.

Only one case control study has looked at anti-angiogenic biomarkers of PE amongst women with SDB, with Bourjeily and colleagues [3] showing that sFlt-1/PlGF ratio, which is highly predictive of PE, is altered in pregnant women with SDB. Despite studies outside pregnancy also demonstrating increased sFlt-1 and sEng in the circulation of patients with SDB, [33,34] we found no differences in any biomarkers between those with or without SDB, in control women or those with established hypertensive disease in pregnancy. Circulating levels of ET-1 have been found to be elevated in preeclamptic pregnancies [35,36] along with non-pregnant cohorts diagnosed with SDB [37]; again we found no effect of SDB. This discordance in results is likely due to the moderate to severe phenotype of SDB in these prior studies compared to our overall low severity of disease.

A recent study found robust evidence for a relationship between SDB measured in early and mid-pregnancy and HDP. [2] They specifically found that 92% of the hypertension diagnoses were made more than 2 weeks after the midpregnancy sleep study, supporting the suggestion that SDB was independently associated with the development of PE. [38,39] Yet, no studies have looked into the diagnostic and pathophysiologic features of gestational hypertensive disorders associated with SDB. Our in-depth results did not support a detrimental role for SDB in the development of hypertensive disease during pregnancy. The most pertinent possibility for a lack of association relates to the phenotype of both SDB and PE featured in our study. The overall degree of SDB diagnosed in our sample was predominantly mild, with a low number of hypoxemic episodes experienced by the mothers. This is important given that shared pathophysiology is predicated on the consequences of intermittent hypoxia following apnea. Although the study was not powered adequately for such analyses, it is worth noting that an association between more severe SDB and adverse outcomes was also lacking in supplementary analyses.

Whilst some studies concentrated on women with severe PE or those admitted and potentially requiring imminent delivery, [11,40,41] we had participants with a range of PE severity, with both a maternal or fetal disease dominating the diagnosis. For example, we have previously reported a woman diagnosed with early-onset PE at 30 weeks gestation who was confirmed to have very severe SDB (RDI/hr = 149). [42] In this case of significant disease, treatment with CPAP resulted in a reduction of the circulating anti-angiogenic factors sFlt-1 and sEng, and stabilisation of ET-1, which paralleled improvement in both clinical and biochemical measures of PE. It may be that this biologically plausible link needs to be explored further within a larger cohort with more severe levels of disease. Nevertheless, our study would caution against universal screening for mild SDB with the view to initiating treatment within HDP, as our data suggest that this low level of disease is unlikely to have a significant impact on perinatal outcomes.

### Blood pressure in pregnancy and the impact of sleep-disordered breathing

In contrast to expectation, the presence of SDB did not contribute to increases in BP across hypertensive pregnancies nor controls–in fact the women with SDB in the GH group had lower SBP and DBP on average. Our results are interesting given the wealth of literature supporting a relationship between SDB and diagnosis of hypertension in the non-pregnant population, [43–46] and the emerging evidence within pregnancy. [2,38,47] Patients with SDB spend their sleep periods in a state of intermittent hypoxia and a cyclic pattern of recurrent surges of vasoconstriction. Repetitive hypoxic stress can alter sympathetic chemoreflex function in patients with OSA, contributing to increased BP during the daytime. [48] Patients with OSA may also develop changes in their autonomic regulation of BP consistent with adaptation of the baroreceptors to higher BP set points. [49–51] While it is intriguing to consider whether the increased sympathetic activity of SDB could be a mechanism for vasoconstriction in some pregnant women [52] contributing to the development of GH or PE, our data did not support this. One key factor for our lack of association between SDB and BP control could relate to pregnancy being a transient condition of only nine months; short duration exposure to the transient respiratory changes in pregnancy may be insufficient to result in downstream physiological effects.

### Strengths and limitations

Our study is amongst the first to look longitudinally at how SDB impacts on diagnosis and severity of hypertensive disease in pregnancy. Most studies to date have simply compared the presence of GH and PE in those with versus without SDB. [2,8,9,38,53] We are also the first study to look at anti-angiogenic markers in a HDP cohort with SDB. The use of full PSG to measure SDB was a key strength of our study over others with abbreviated monitoring techniques. This allowed us to most accurately designate pregnant women by SDB status, and to include subtle respiratory events associated with cortical arousal to be included when calculating severity of disordered breathing. Our use of EEG monitoring is likely a key factor behind the SDB prevalence discrepancy between the largest study of SDB during pregnancy to date (8.3%) [2] and this study (39–63%), along with differences in gestation and BMI.

The most notable limitation of our study is sample size. The power calculation was sufficient for our primary study which compared the frequency of SBD in HDP to normotensive pregnancy, [10] however the decision to split the GH and PE participants due to their very different pathophysiology means the results need to be interpreted with caution. Due to the logistics of obtaining the maternal blood samples at delivery, we unfortunately collected 21 blood samples in the third trimester that did not have a matching delivery sample. Also, timing of blood sample collection in the third trimester and delivery was quite variable, mostly as GH and PE participants typically delivered their babies sooner resulting in a shorter time lapse between samples. Nevertheless, we attempted to overcome this by calculation of a ‘change per week’ assessment for anti-angiogenic markers.

From a statistical perspective, we were only able to measure SDB status at one time-point during pregnancy which is not ideal for survival analysis and mixed modelling. We know that severity of SDB can change across pregnancy however the magnitude of this is minor. [53,54] Allocating SDB status as measured in the third trimester via PSG was our best option and it is unlikely that this significantly affected our results. The other limitation of performing the PSG at one time point is the ability to infer causality. The majority of the literature points to SDB having a downstream effect on cardiovascular outcomes including hypertension. In our study we performed PSG after the HDP diagnosis, however we cannot be sure whether the HDP or SDB came first and so our results could be interpreted to infer that those with more severe HDP do not have a higher propensity to develop SDB.

Our study design excluded women with a pre-existing sleep disorder including SDB. Interestingly, in the recruitment process for this study only one woman was excluded due to a prior diagnosis of SDB. Conversely, 17.6% of women in our sample had moderate-to-severe SDB. It is likely their SDB preceded pregnancy and they were not aware of it, or not sufficiently concerned to seek medical investigation. It remains unclear whether pre-existing SDB or the development of SDB in pregnancy is more problematic for pregnancy outcomes, [55–57] however this exclusion criteria was unlikely to impact our results.

## Conclusion

Our study is amongst the first to look specifically at how SDB impacts on clinical, biochemical and anti-angiogenic measures of hypertensive disease in pregnancy. We found that the presence of SDB overall did not influence the course of hypertensive disease in pregnancy. Given the number of studies confirming the relationship between SDB and diagnosis of HDP, the causal pathways still require further study. Better understanding of this relationship will be informed by future research focusing on more severe levels of SDB, and with SDB pre-dating pregnancy. Nevertheless, cause and effect will always be difficult to completely disentangle, particularly given the potential contribution of residual confounders, particularly obesity. Ultimately, the impact of SDB on pregnant women and their infants may only be determined through an interventional treatment trial, by measuring its success in prolonging pregnancies affected by hypertensive disorders.

## Supporting information

S1 Table Indices of severity of hypertensive disease for each group stratified by SDB status defined as RDI ≥ 15. (DOCX)

S2 Table Change per week for anti-angiogenic markers of hypertensive disease in pregnancy with SDB defined as RDI ≥ 15. (DOCX)
