# Supplementary material for: Sleep-disordered breathing does not impact maternal outcomes in women with hypertensive disorders of pregnancy
Source: PLoS One. 2020 Apr 27;15(4):e0232287. doi: 10.1371/journal.pone.0232287 (PMC7185691; doi:10.1371/journal.pone.0232287)
Supplement: S1 Table — (DOCX) [file pone.0232287.s001.docx]

Table S1 *Indices of Severity of Hypertensive Disease for Each Group Stratified by SDB Status Defined as RDI* ≥ *15*

|  | PE (n = 17) | |  | GH (n = 24) | |  | Controls (n = 44) | |  |
| --- | --- | --- | --- | --- | --- | --- | --- | --- | --- |
|  | RDI ≥ 15  (n = 4) | No SDB  (n = 13) | p | RDI ≥ 15  (n = 5) | No SDB  (n = 19) | p | RDI ≥ 15  (n = 6) | No SDB  (n = 38) | p |
| RDI | 74.2 (28.3, 129.6) | 1.9 (1.7, 4.6) | .003 | 35.6 (26.1, 55.8) | 5.1 (3.0, 9.1) | <.001 | 36.7 (24.4, 83.6) | 3.9 (2.2, 5.5) | <.001 |
| Gestation Diagnosis (weeks) | 31.4 (29.9, 33.4) | 29.0 (26.6 – 32.3) | .43 | 34.3 (33.9, 36.4) | 33.4 (30.7 – 34.6) | .14 | - | - | - |
| Diagnosis to Delivery (days)* | 35.0 (1.0, 46.0) | 11.0 (9.0, 17.0) | .38 | 23.0 (15.0, 35.0) | 34.0 (20.0, 47.0) | .46 | - | - | - |
| Gestation Delivery (weeks)* | 37.1 (34.9, 37.3) | 30.0 (28.3 – 35.1) | .04 | 38.4 (37.9, 38.6) | 37.9 (36.7, 38.9) | .39 | 38.6 (38.3, 39.4) | 39.4 (38.4, 40.7) | .31 |
| Early Onset (<34 weeks) | 3 (75.0%) | 11 (84.6%) | 1.0 | - | - |  | - | - | - |
| FGR with PE diagnosis | 1 (25.0%) | 9 (69.2%) | .25 | - | - |  | - | - | - |
| Severe HTN*^#^* | 2 (50.0%) | 10 (76.9%) | .54 | 0 (0.0%) | 7 (36.8%) | .27 | - | - | - |
| % Antihypertensive | 3 (75.0%) | 11 (84.6%) | 1.0 | 3 (60.0%) | 17 (89.5%) | .18 | 0 (0%) | 1 (2.7%) |  |
| >1 Antihypertensive | 2 (50.0%) | 8 (61.5%) | 1.0 | 0 (0.0%) | 3 (15.8%) | 1.0 | - | - |  |
| Gestation Started Antihypertensives (weeks) | 32.1 (24.7 – 34.9) | 31.7 (27.3 – 33.9) | .93 | 36.4 (34.4 – 37.6) | 34.0 (31.1 – 36.6) | .29 | - | - |  |
| Developed PE | - | - |  | 2 (40.0%) | 6 (31.6%) | 1.0 | 0 (0%) | 0 (0%) | - |
| Developed GH | - | - |  | - | - |  | 0 (0.0%) | 3 (7.9%) | 1.0 |
| *Biochemical and Haematological Markers* | | | | | | |  |  |  |
| Peak Pr:Cr Ratio | .12 (.06 - .28) | .12 (.05 - .39) | .96 | .03 (.03 - .06) | .02 (.02 - .06) | .30 | - | - |  |
| Peak ALT | 25.5 (13.5 – 56.3) | 23.0 (16.0 – 40.0) | 1.0 | 20.0 (13.0 – 33.0) | 23.0 (17.0 – 37.0) | .58 | - | - |  |
| Peak Urate | 0.49 (0.36 – 0.56) | 0.49 (0.35 – 0.51) | .86 | 0.31 (0.31 – 0.34) | 0.35 (0.32 – 0.40) | .14 | - | - |  |
| Peak Creatinine | 62.0 (59.0 – 81.5) | 72.0 (63.0 – 81.0) | .82 | 57.0 (49.0 – 71.0) | 63.0 (52.0 – 64.0) | .94 | - | - |  |
| Nadir Platelets | 186.5 (178.0 – 240.0) | 187.0 (151.0 – 216.0) | .65 | 248.0 (233.0 – 260.0) | 178.0 (157.0 – 229.0) | .055 | - | - |  |
| Peak Antenatal BP  Systolic mmHg  Diastolic mmHg | 161.3 ± 15.3  103.8 ± 6.2 | 166.6 ± 10.1  102.4 ± 5.0 | .42  .66 | 154.8 ± 4.4  98.8 ± 6.6 | 158.4 ± 11.1  101.7 ± 6.4 | .50  .38 | 126.8 ± 9.3  79.2 ± 7.6 | 128.3 ± 10.1  80.0 ± 7.4 | .75  .80 |

*Note*. Values given as Mdn (IQR) or n (%). SBD = sleep-disordered breathing, RDI = respiratory disturbance index, PE = preeclampsia, GH = gestational hypertension, FGR = fetal growth restriction, HTN = hypertension, Antihypert = antihypertensive, Pr:Cr = protein:creatinine, ALT = alanine transaminase, BP = blood pressure.

*Three CPAP users removed from analysis for this outcome.

^#^ defined as systolic BP ≥ 160mmHg and/or diastolic BP ≥ 110mmHg
