# Supplementary material for: Sleep-disordered breathing does not impact maternal outcomes in women with hypertensive disorders of pregnancy
Source: PLoS One. 2020 Apr 27;15(4):e0232287. doi: 10.1371/journal.pone.0232287 (PMC7185691; doi:10.1371/journal.pone.0232287)
Supplement: S2 Table — (DOCX) [file pone.0232287.s002.docx]

Table S2 *Change per Week for Anti-Angiogenic Markers of Hypertensive Disease in Pregnancy with SDB Defined as RDI* ≥ *15*

|  | HDP  (n = 23)* | |  | Controls  (n = 24) | |  |
| --- | --- | --- | --- | --- | --- | --- |
|  | RDI ≥ 15  (n = 6) | No SDB  (n = 17) | p | RDI ≥ 15  (n = 4) | No SDB  (n = 20) | p |
| RDI | 37.7 (25.3, 59.2) | 3.5 (1.9, 7.5) | <.001 | 27.3 (20.3, 39.9) | 3.8 (2.1, 4.6) | <.001 |
| ET-1 (pg/ml) | -0.04 (-0.21, 0.14) | 0.20 (-0.13, 0.66) | .29 | 0.09 (0.04, 0.29) | 0.05 (-0.04, 0.14) | .35 |
| sEng (ng/ml) | 0.7 (-0.1, 3.8) | -0.3 (-1.1, 4.2) | .56 | 0.3 (0.0, 1.0) | 0.7 (0.2, 1.9) | .31 |
| sFlt-1 (pg/ml) | 679.2 (292.8, 1790.3) | 553.3 (-181.5, 1184.6) | .56 | 153.4 (57.6, 323.4) | 394.2 (275.0, 717.1) | .06 |
| PlGF (pg/ml) | -14.9 (-23.0, 4.6) | -4.1 (-14.4, 0.0) | .56 | -22.6 (-38.5, -4.7) | -13.7 (-32.1, -6.1) | .85 |
| sFlt-1/PlGF | 8.2 (-2.6, 24.7) | 10.2 (1.7, 34.0) | .73 | 1.0 (0.6, 4.3) | 7.8 (2.9, 12.4) | .07 |

*Note.* Values given as Mdn (IQR). RDI = respiratory disturbance index, HDP = hypertensive disorders of pregnancy, SDB = sleep-disordered breathing, ET-1 = endothelin-1, sEng = soluble endoglin, sFlt-1 = soluble fms-like tyrosine kinase-1, PlGF = placental growth factor.

Three participants on CPAP excluded.

*One PE participant was excluded from analysis due to extreme outlying values (z scores > 5.3).
